# Analysis of tumor microenvironment alterations in partially responsive rectal cancer patients treated with neoadjuvant chemoradiotherapy

**DOI:** 10.1007/s00384-024-04672-1

**Published:** 2024-06-26

**Authors:** Hong Chen, Ji-Hong Zhang, Qin Hao, Xin-Lin Wu, Jia-Xing Guo, Cong-Xiu Huang, Jun Zhang, Guo-Sheng Xing, Zhi-Lin An, Yu Ling, Jian-Guo Zhao, Ying-Na Bao

**Affiliations:** 1 Affiliated Hospital of Inner Mongolia Medical University, Hohhot, 010050 China; 2 Department of Radiotherapy, Affiliated Hospital of Inner Mongolia Medical University, Hohhot, 010050 China; 3 Department of Gastrointestinal Surgery, Affiliated Hospital of Inner Mongolia Medical University, Hohhot, 010050 China

**Keywords:** Neoadjuvant chemoradiotherapy, Tumor microenvironment, Locally advanced rectal cancer, Partial response, Immunostimulation, Immunosuppression

## Abstract

**Purpose:**

Achieving a pathologic complete response (pCR) after neoadjuvant chemoradiotherapy (NCRT) remains a challenge for most patients with rectal cancer. Exploring the potential of combining NCRT with immunotherapy or targeted therapy for those achieving a partial response (PR) offers a promising avenue to enhance treatment efficacy. This study investigated the impact of NCRT on the tumor microenvironment in locally advanced rectal cancer (LARC) patients who exhibited a PR.

**Methods:**

This was a retrospective, observational study. Five patients demonstrating a PR after neoadjuvant treatment for LARC were enrolled in the study. Biopsy samples before treatment and resected specimens after treatment were stained with a panel of 26 antibodies targeting various immune and tumor-related markers, each labeled with distinct metal tags. The labeled samples were then analyzed using the Hyperion imaging system.

**Results:**

Heterogeneity within the tumor microenvironment was observed both before and after NCRT. Notably, tumor-associated macrophages, CD4 + T cells, CD8 + T cells, CD56 + natural killer cells, tumor-associated neutrophils, cytokeratin, and E-cadherin exhibited slight increase in abundance within the tumor microenvironment following treatment (change ratios = 0.78, 0.2, 0.27, 0.32, 0.17, 0.46, 0.32, respectively). Conversely, the number of CD14 + monocytes, CD19 + B cells, CD45 + CD4 + T cells, collagen I, α-smooth muscle actin, vimentin, and β-catenin proteins displayed significant decreases post-treatment (change ratios = 1.73, 1.92, 1.52, 1.25, 1.52, 1.12, 2.66, respectively). Meanwhile, Foxp3 + regulatory cells demonstrated no significant change (change ratio = 0.001).

**Conclusions:**

NCRT has diverse effects on various components of the tumor microenvironment in LARC patients who achieve a PR after treatment. Leveraging combination therapies may optimize treatment outcomes in this patient population.

**Supplementary Information:**

The online version contains supplementary material available at 10.1007/s00384-024-04672-1.

## Background

Rectal cancer represents one of the prevalent malignant tumors affecting the digestive tract and is often diagnosed at a locally advanced stage [1]. The effectiveness of neoadjuvant chemoradiotherapy (NCRT) in reducing the tumor stage and local recurrence rates has been well established. Consequently, the integration of NCRT with total mesorectal excision has evolved into the standard treatment strategy for patients diagnosed with locally advanced rectal cancer (LARC) [2]. However, clinical studies have demonstrated that approximately 40% of LARC patients exhibit a partial response (PR) following NCRT, with only 8 to 20% achieving a pathologic complete response (pCR) after surgery [3]. The low pCR rate in the management of LARC represents a critical issue that demands urgent attention. In light of this challenge, it is imperative to explore and develop innovative treatment strategies, including immunotherapy and targeted therapy, aimed at transitioning a larger proportion of PR patients into pCR patients, beyond what conventional chemoradiotherapy (CRT) can achieve.

The tumor microenvironment is a comprehensive ecosystem comprised of various components, including tumor cells, endothelial cells, immune cells, fibroblasts, smooth muscle cells, adipocytes, and other stromal cells. It represents a crucial focal point for research in the field of tumor therapy [4]. Many studies have reported the interaction between CRT and the tumor microenvironment [5, 6]. Building upon these research findings, a range of integrated treatment strategies have been developed. Immunotherapy is a therapeutic approach that harnesses and amplifies the activity of anti-tumor effector cells, and its effectiveness is intricately linked to the dynamics of the tumor microenvironment. Additionally, radiotherapy serves to facilitate the release and presentation of tumor cell antigens, augment the infiltration of CD8 + T cells within the tumor microenvironment, and activate the anti-tumor immune response [7]. The combination of these two approaches works synergistically to enhance the body’s anti-tumor immunity, resulting in a more favorable clinical outcome [8]. However, radiation can have a negative impact on the tumor microenvironment by promoting the accumulation of regulatory cells (Tregs), myeloid-derived suppressor cells, and tumor-associated macrophages (TAMs). This can contribute to the formation of an immunosuppressive tumor microenvironment, which plays a role in therapeutic resistance [9]. In such scenarios, the combination of targeted therapy with conventional CRT has emerged as a promising approach. Multiple preclinical studies have shown that when CRT is paired with the blockade of immunosuppressive cells, like Tregs and TAMs, it fosters anti-tumor immunity and enhances the infiltration of cytotoxic T cells. This combination therapy has demonstrated a superior capacity to inhibit tumor growth compared to monotherapy [10, 11].

Currently, combination therapies targeting the tumor microenvironment cellular components have been shown to help improve tumor outcomes. To increase the rate of conversion from PR to complete response (CR) in patients with LARC and to provide guidance for future research on the integration of CRT, immunotherapy, and targeted therapies, it is crucial to elucidate how CRT impacts the tumor microenvironment in LARC patients who achieve a PR after treatment. However, it is worth noting that the majority of clinical studies examining the influence of NCRT on the tumor microenvironment in LARC patients often lack patient categorization based on therapeutic sensitivity at enrollment and tend to encompass a broad spectrum of LARC patients [12, 13]. In addition, the scant studies focusing on the tumor microenvironment before and after NCRT predominantly concentrate on selected immune cell types. Additionally, the detection methods employed often rely on traditional research techniques, like immunohistochemistry. As a result, investigations into the changes within the tumor microenvironment before and after NCRT remain relatively limited in scope and depth [14, 15]. The Hyperion imaging system represents an advanced instrument that offers unparalleled capabilities in both ultra-high-channel flow and tissue imaging. It represents a potent tool for conducting research on the tumor microenvironment. To comprehensively and precisely evaluate the impact of NCRT on the tumor microenvironment in LARC patients, we employed the Hyperion imaging system for the very first time in this area. This innovative approach allowed us to examine the tumor microenvironment both before and after NCRT in five LARC patients who had achieved a PR following treatment. Through this investigation, we were able to delineate the alterations induced by NCRT on the constituent cells within the tumor microenvironment during the course of treatment. Subsequently, we put forth a mechanistic understanding of how NCRT shapes the tumor microenvironment, and thus influences therapeutic efficacy and patient prognosis.

## Methods

### Patients

We conducted a retrospective observational study of patients with LARC who were treated at the Department of Radiotherapy of the Affiliated Hospital of Inner Mongolia Medical University, between October 2020 to April 2021. To enhance the homogeneity of the patient cohort, the inclusion criteria encompassed the following: (1) aged between 50 and 80 years old, (2) absence of prior abdominal or pelvic radiotherapy, (3) histopathological confirmation of adenocarcinoma, (4) T3–4 staging or node-positive disease without distant metastases, (5) achievement of PR following NCRT, (6) Karnofsky performance score ≥ 70, (7) undergoing surgical resection subsequent to NCRT, and (8) absence of a history of other malignancies. All patients underwent a course of treatment consisting of five weeks of intensity-modulated radiotherapy or tomotherapy, involving 27 fractions of 49.95 Gy each delivered to the pelvic region. A supplementary dose of 4.05 Gy was applied to the primary gross tumor. Concurrently, patients received chemotherapy with capecitabine (825 mg/m^2^) twice daily for 21 days. The evaluation of tumor response and surgical procedures were carried out 8–12 weeks after completing concurrent CRT. Tissue samples for analysis were obtained from pre-NCRT biopsies and post-NCRT surgical specimens of the LARC patients, with the section thickness set at 2 µm.

This research was conducted in accordance with the approval of the ethics committee of the Affiliated Hospital of Inner Mongolia Medical University (Approval No: 2021 Drug No. (043)). The study adhered to the principles outlined in the Declaration of Helsinki, as revised in 2013. All participants provided written informed consent before their inclusion in the study. Also, our study adhered to the STROBE (strengthening the reporting of observational studies in epidemiology) statement.

### Assessment of PR and follow-up

PR was primarily evaluated through pelvic magnetic resonance imaging and abdominal computed tomography. The diagnosis of PR was determined in accordance with the Response Evaluation Criteria in Solid Tumors 1.1 [16], where PR is defined as a reduction of at least 30% in the sum of the diameters of the target lesions from the baseline, although complete disappearance has not been achieved. Follow-up was conducted in outpatient clinics and by telephone, with the last follow-up of this cohort conducted on July 7, 2023.

### Panel and antibody conjugation

For this study, an antibody panel was specifically designed, featuring a total of 20 immune-related antibodies and six antibodies targeting tissue structural proteins. This panel allowed distinguishing between various immune cell types, intercellular components, and tumor cells within the tumor microenvironment of LARC. Each of these antibodies was linked to metal tags, which possessed the capability to label both the cell surface and intracellular proteins. As illustrated in Supplementary Table 1, CD antibodies and structural proteins were employed to visualize the immune cells and key histological landmarks.

### Hyperion tissue image scanning

In the scanning module, a high-precision laser ablation system was used to point-by-point scan the marked tissue section samples. The maximum square area scanned was 500 × 500 µm, and the laser beam cross-section was 1 × 1 µm. Images were acquired and analyzed using the Hyperion imaging system (Fluidigm) [17].

### Data procedure and normalization

Commercial acquisition software (MCD viewer software) was used to output the original scan files (MCD files) as individual tiff files for each antibody. Meanwhile, the image module in Python was used to identify the tiff files and remove background noise. CellProfiler software was used to identify the region where the nucleus was located and the cell boundaries to locate the individual cell locations [18]. HistoCAT software was used to quantify each antibody in the cells, and the antibody values in a single image [19]. The cell subsets were then identified, and the individual antibody scores from each slide were normalized using quantile normalization (0.95) to reduce the effects between batches. For t-SNE and PhenoGraph, the data were normalized using Harmony imaging and analysis software [20]. High-dimensional data were reduced to two dimensions for visualization using the t-SNE algorithm [21]. Individual cells were clustered into groups based on their phenotypic similarity and molecular markers using PhenoGraph [22]. The *T*-test was used to compare the changes of cellular antibody expression before and after treatment in the five patients.

## Results

### Preprocessing the LARC samples and data acquisition

**Fig. 1:**
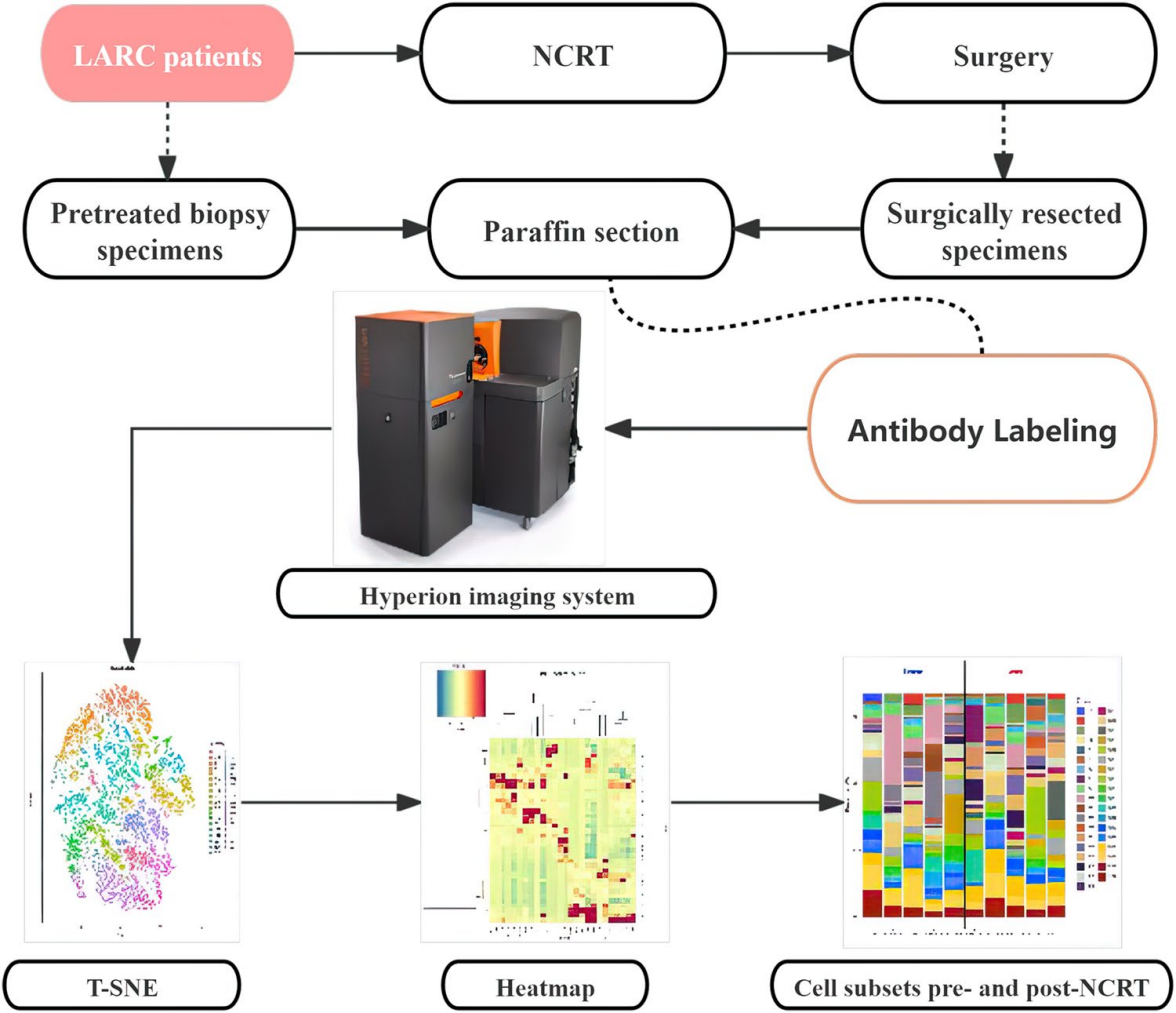
Flow diagram for the study

**Fig. 2:**
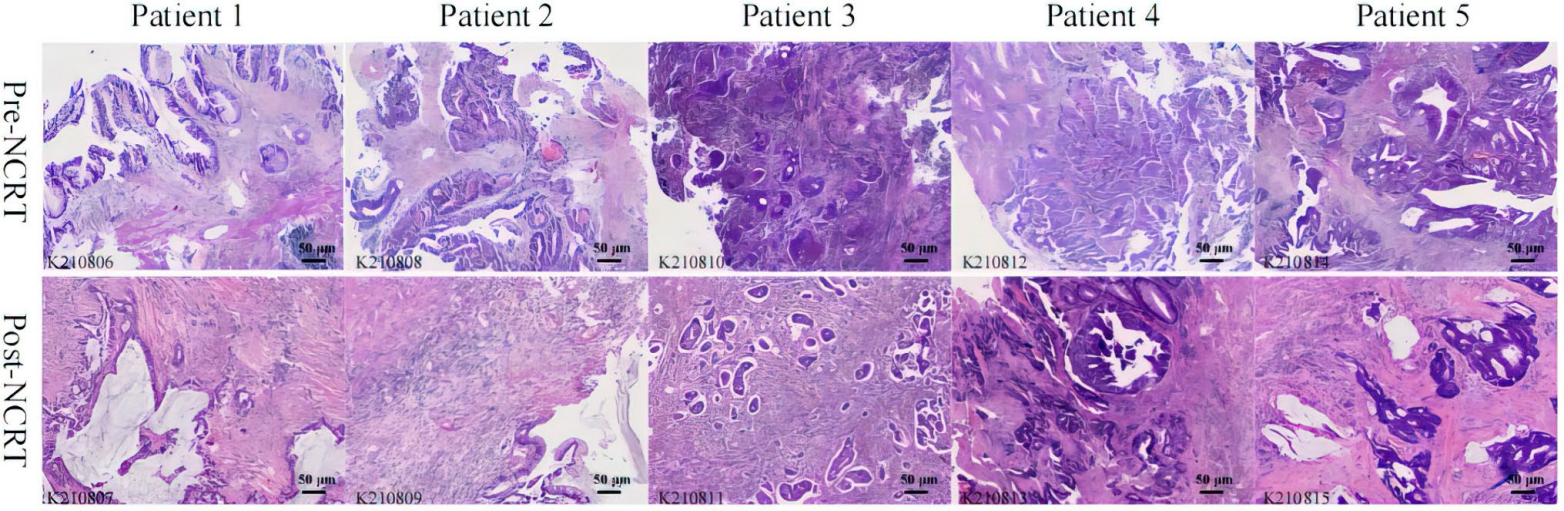
Paraffin sections of the five patients with locally advanced rectal cancer(LARC) pre-treatment (*n* = 5) and post-treatment (*n* = 5). K2108XX is the picture number. Representative image scale bars = 50 μm

After a thorough screening process of the LARC patients who had undergone NCRT at the Department of Radiotherapy, Affiliated Hospital of Inner Mongolia Medical University, between October 2020 to April 2021, a total of five patients were identified as meeting our clinical inclusion criteria. Their clinical characteristics are summarized in Table 1. These patients were diagnosed with rectal adenocarcinoma at the T3-4NxM0 stage, and a uniform treatment approach was applied to all, resulting in the achievement of a PR following NCRT. The flow diagram for the study design is shown in Fig. 1. Paraffin-embedded tissue sections were collected from five LARC patients both before and after NCRT. In total, ten paraffin sections were prepared as experimental samples and individually labeled with a predefined antibody panel. The labeled samples were subsequently identified and analyzed using the Hyperion imaging system, as depicted in Fig. 2.

**Table 1:** Clinicopathological characteristics of the five included patients with locally advanced rectal cancer (LARC)

Study no	Gender	Age (years)	Pathological*	Distance from anal verge (cm)	TNM stage	Treatment	Response evaluation	Follow-up (months)
Patient 1	Male	58	as-ca	> 5	cT3N0M0	CRT + TME	PR	No recurrence, no metastasis, 31 months
Patient 2	Male	78	as-ca	≤ 5	cT3N1M0	CRT + TME	PR	No recurrence, no metastasis, 31 months
Patient 3	Male	55	as-ca	> 5	cT3N2M0	CRT + TME	PR	No recurrence, no metastasis, 30 months
Patient 4	Male	67	as-ca	> 5	cT4N1M0	CRT + TME	PR	Metastasis, 29 months
Patient 5	Male	69	as-ca	> 5	cT4N1M0	CRT + TME	PR	Death, 25 months

^*^as-ca = adenocarcinoma

### Analysis of the tumor microenvironment pre- and post-NCRT of LARC using the Hyperion imaging system

**Fig. 3:**
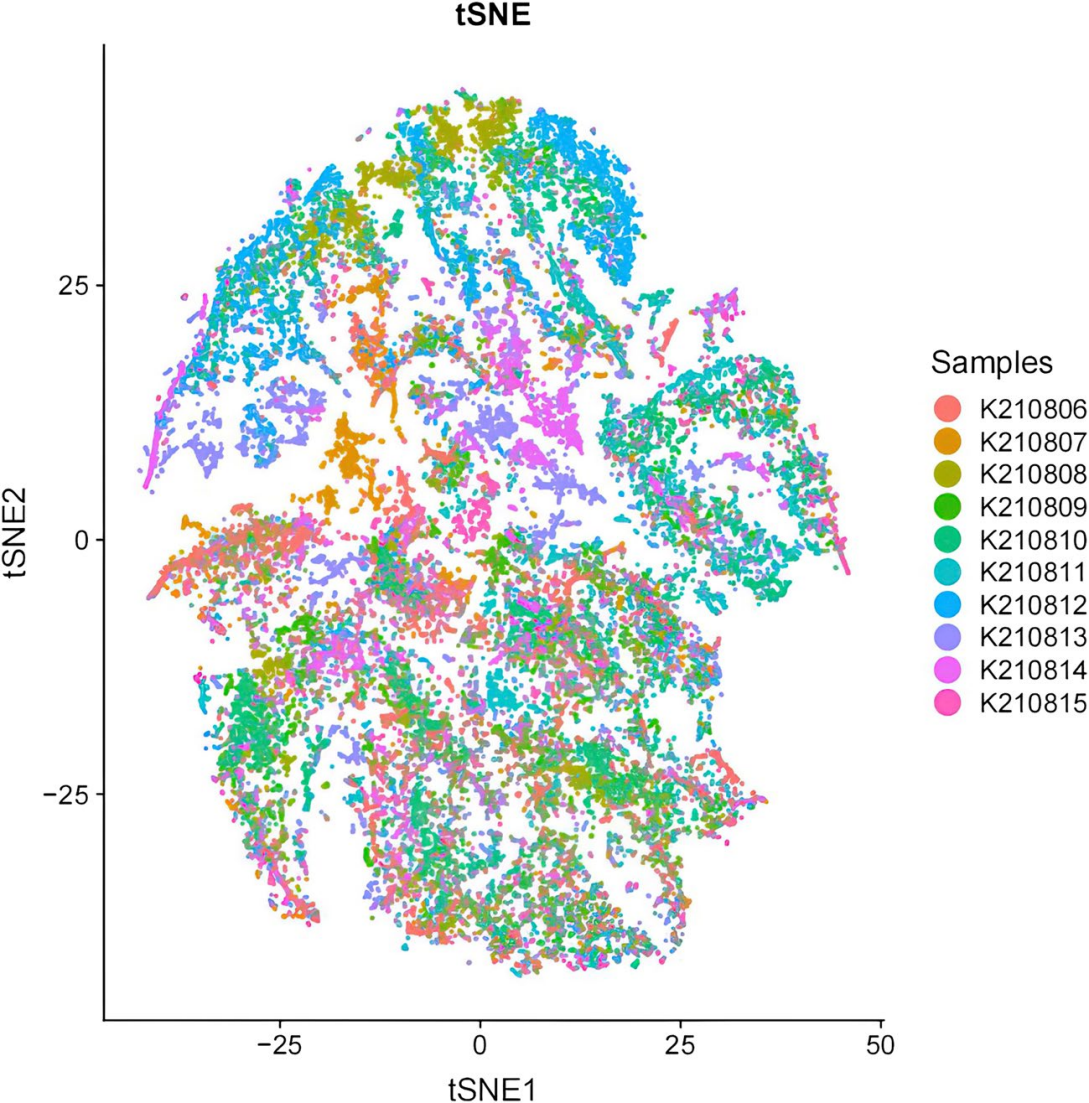
t-SNE descending dimension map of 10 locally advanced rectal cancer(LARC) samples. Colors represent different samples

To investigate whether NCRT could bring about changes in the tumor microenvironment, we employed t-SNE analysis to visualize the tumor microenvironment before and after NCRT across all the LARC patients (Fig. 3). Our observations revealed that the cell subsets before and after NCRT did not entirely overlap; instead, independent cell subsets were discernible, signifying the inherent heterogeneity of the tumor microenvironment in the LARC patients before and after NCRT.

**Fig. 4:**
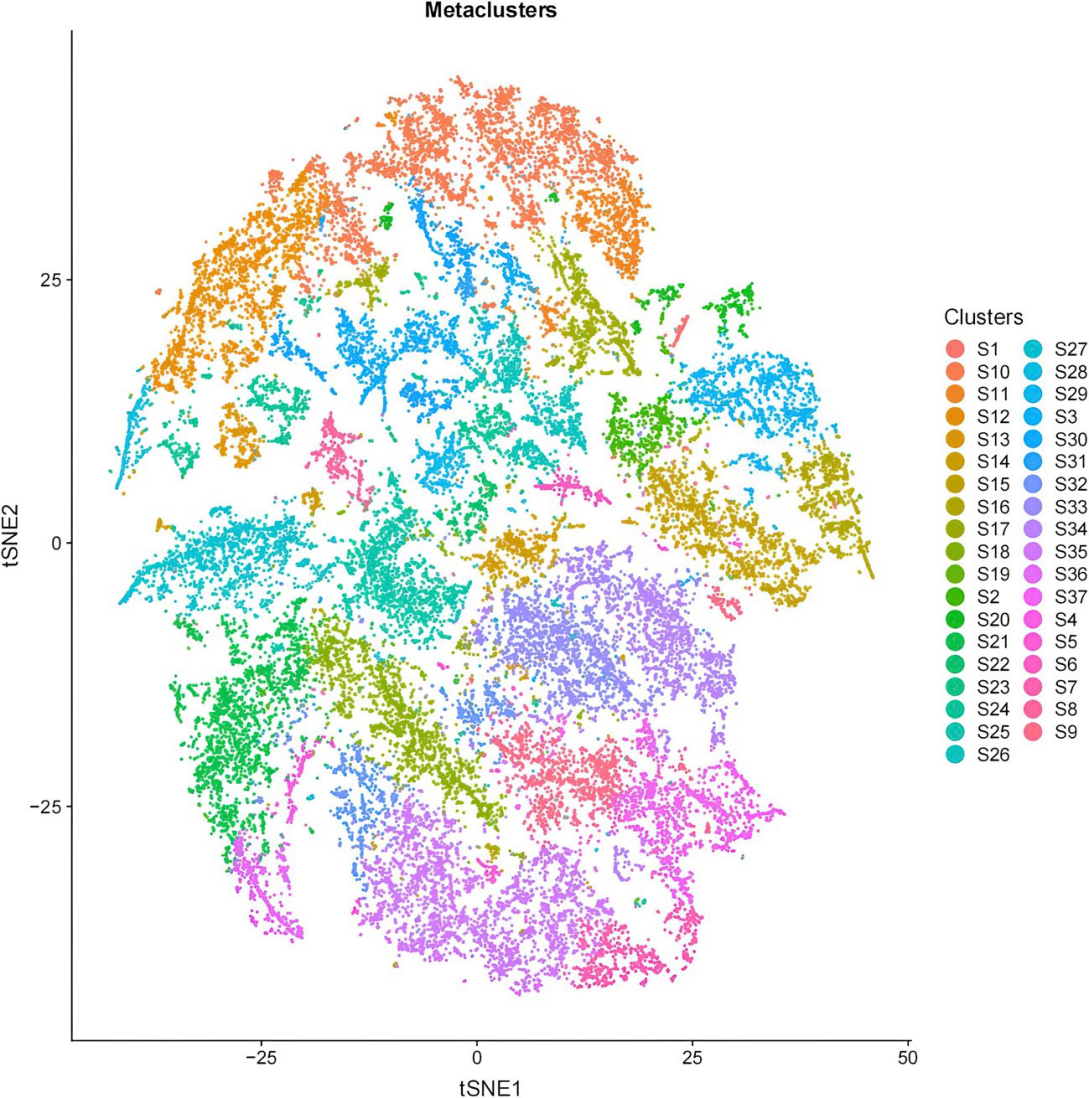
t-SNE visualization of locally advanced rectal cancer (LARC) cell clusters. Colors represent different cell clusters

**Fig. 5:**
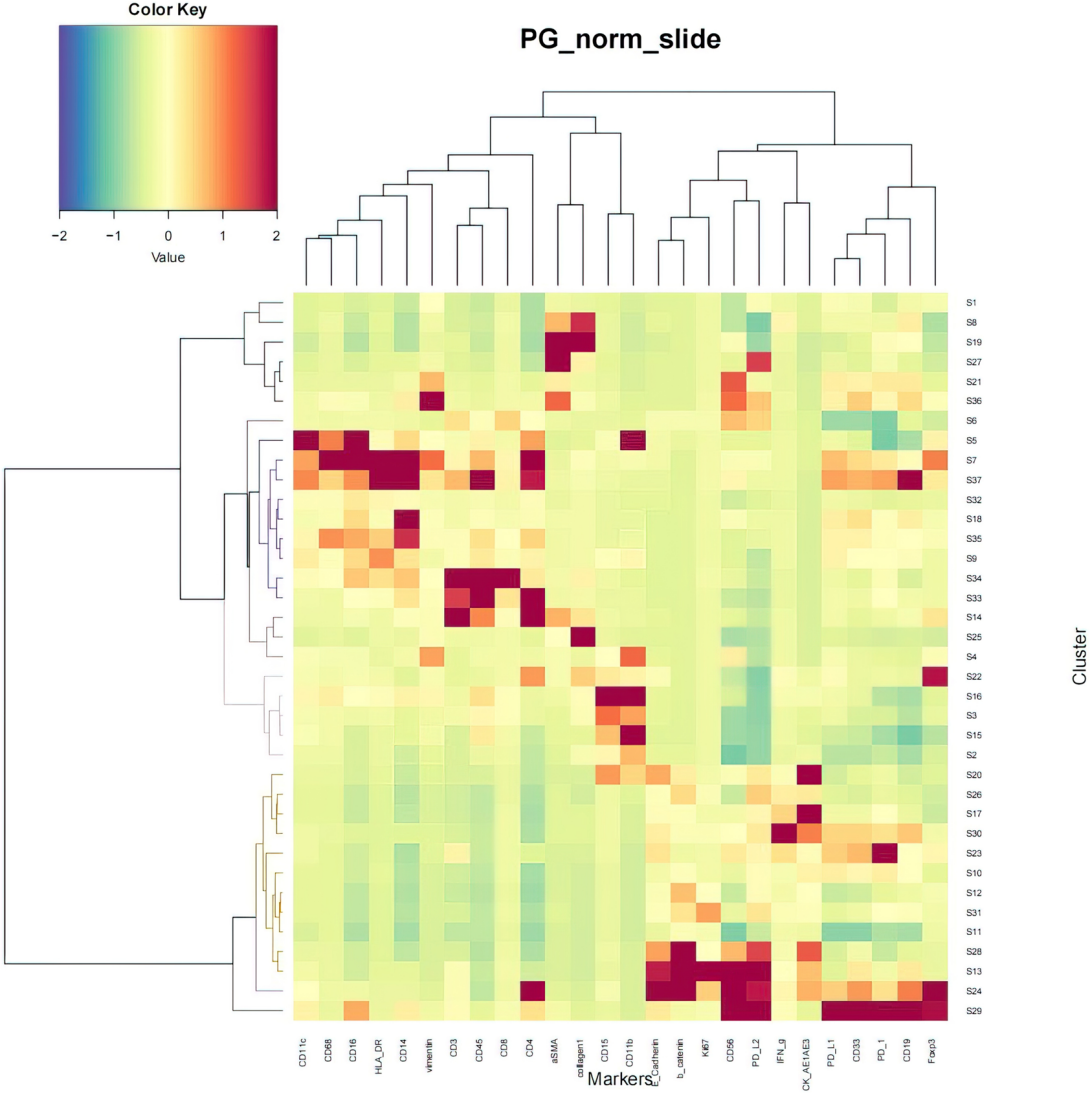
Heat map of locally advanced rectal cancer (LARC) samples. The heat map shows the differential expression of tagged antibodies in 37 cell subsets. According to the typically expressed markers, some cell clusters are identified as known cell types

Subsequently, a t-SNE map was generated based on various clusters of cell subsets to provide an overview of the immune cell subsets and the extracellular matrix in the tumor microenvironment before and after NCRT in the LARC patients (Fig. 4). Within this panoramic representation, we identified 37 distinct cell subsets in the tumor microenvironment, categorized according to their phenotypic similarities. Heat maps were then constructed, reflecting the calculated antibody expression levels within each of these cell subpopulations (Fig. 5). These 37 different cell subsets encompassed various macrophage subsets, CD56 + natural killer (NK) cell subsets, CD4 + T cell subsets, CD8 + T cell subsets, CD15 + granulocyte subsets, CD14 + monocyte subsets, CD19 + B cell subsets, and Foxp3 + Tregs cell subsets, along with structural proteins, such as collagen I, α-smooth muscle actin (α-SMA), vimentin, and β-catenin, and cell subsets related to E-cadherin and cytokeratin (CK).

Alterations in cell subsets in the LARC patients pre- and post-NCRT and their implications.

## Alterations in cell subsets in the LARC patients pre- and post-NCRT

**Fig. 6:**
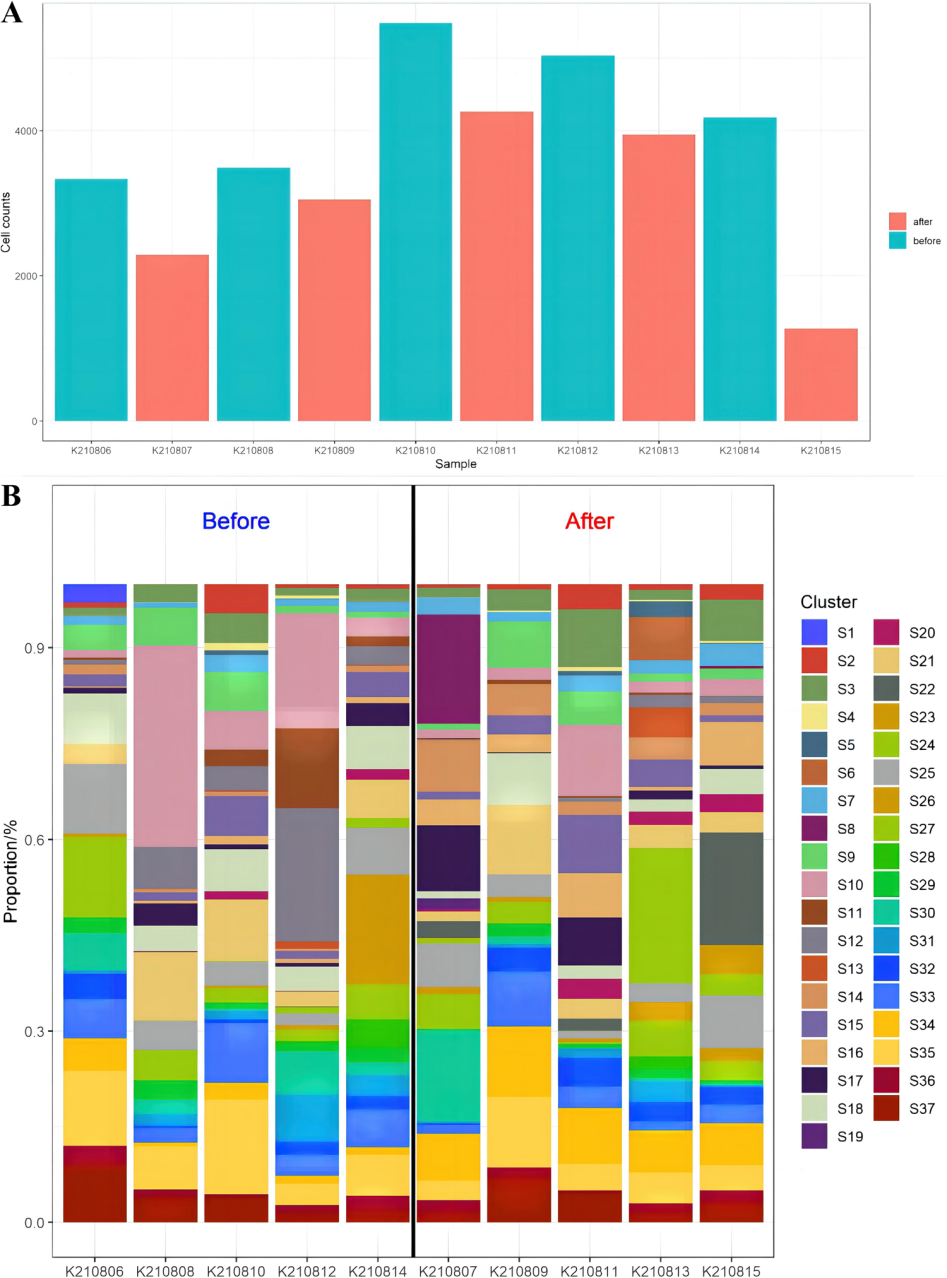
Changes in the tumor microenvironment in locally advanced rectal cancer (LARC). **A** Cell count changes of each patient before and after neoadjuvant chemoradiotherapy (NCRT); blue represents before treatment, and red represents after treatment. The abscissa is the patient number, and the ordinate is the cell count. **B** Changes in cell subsets in each patient before and after NCRT, with different colors representing different cell subsets. The abscissa is the patient number, and the ordinate is the proportion of cell subsets

Our analysis demonstrated significant post-treatment increases in all antibodies within the tumor microenvironment, with the exception of CD19 and PD-L2, in the five LARC patients who achieved a PR (Supplementary Table 2). Conversely, the cell counts in the post-treatment samples generally exhibited a decrease compared to the pre-treatment samples (Fig. 6A). This decline encompassed various cell types, including tumor cells, immune cells, and matrix cells. Notably, CRT induced a partial stimulation of cell surface antigen expression, but the overall cell count decreased due to the effect of the CRT.

While the proportions of each cell subset did not display discernible differences in the pre- and post-treatment samples (Fig. 6B), further analysis revealed varying degrees of change across the 37 cell subsets when calculating the change ratio for all the cell subsets (Supplementary Table 3). For clarity, cell subsets with an insignificant antibody expression were excluded in Supplementary Table 3, leaving two distinct groups: immune cell subsets (Table 2) and histological landmarks (Table 3). In Table 2, it can be seen that there were significant decreases in CD14 + monocytes, CD19 + B cells, and CD45 + CD4 + T cells post-treatment. Simultaneously, a slight increase in the infiltration of CD4 + T cells, CD8 + T cells, CD56 + NK cells, TAMs, and tumor-associated neutrophils (TANs) could be noted, with a relatively low change ratio. Surprisingly, there was no substantial change in the population of Foxp3 + Tregs. These alterations in immune cells suggested that NCRT effectively reduced the infiltration of certain immune cells while minimally impacting the recruitment of immunosuppressive cells and immune activation cells. Moreover, Foxp3 + Tregs appeared to be unaffected by NCRT.

**Table 2:** Clarification of the immune cell subsets

Cell subsets	Related highly expressed antibodies	Fold change
S29	Foxp3, CD19, CD33, CD56, PD_1, PD_L1, PD_L2	1.92
S18	CD14	1.73
S35	CD14	1.59
S33	CD3, CD4, CD45	1.52
S37	CD14, CD19, CD4, CD45, HLA_DR	1.13
S15	CD11b	0.78
S7	CD4, CD14, HLA-DR, CD16, CD68	0.59
S13	CD56, PD-L2, Ki67, b_catenin, E-cadherin	0.32
S5	CD11b, CD16, CD11c	0.31
S34	CD8, CD45, CD3	0.27
S14	CD4, CD3	0.2
S16	CD11b, CD15	0.17
S24	Foxp3, CD4, CD56, PD-L2, b_catenin, E-cadherin	0.11
S22	Foxp3	0.001
S23	PD_1	0

**Table 3:** Histological features of the cell subsets

Cell subsets	Related highly expressed antibodies	Fold change
S17	CK-AE1AE3	0.46
S20	CK-AE1AE3	0.37
S13	CD56, PD-L2, Ki67, b_catenin, E-cadherin	0.32
S19	Collagen1, aSMA	0
S8	Collagen1	0.003
S25	Collagen1	1.25
S27	PD_L2, aSMA	1.52
S28	b_catenin	2.66
S30	IFN_g	1.07
S36	Vimentin	1.12

## Implications of the structural protein changes and prognostic associations

We utilized structural proteins to visualize the histological landmarks and tracked their changes pre- and post-NCRT. The data presented in Table 3 revealed increased levels of E-cadherin and CK after treatment, while vimentin, collagen I, α-SMA, and β-catenin protein exhibited decreased levels. These findings suggest that NCRT may have the capacity to reverse the occurrence of epithelial-mesenchymal transformation (EMT) by significantly reducing the components associated with EMT and increasing the presence of epithelial cells.

Moreover, when comparing the ratio of changes in the increased and decreased cell subsets, it was apparent that the change ratio of the decreased cell subsets after treatment was more extensive. Remarkably, apart from CD45 + CD4 + T cells, the other decreased cell subsets were associated with increased invasiveness and poorer prognosis in colorectal cancer (CRC) [23–27]. Consequently, these findings suggest that NCRT can significantly reduce the number of cells associated with a poor prognosis within the tumor microenvironment of LARC patients who achieve a PR after CRT.

## Discussion

Considering the increasing body of evidence linking immune cell infiltration in the tumor microenvironment to varying treatment responses, our hypothesis posited that patients with diverse treatment sensitivities would exhibit distinct alterations in the tumor microenvironment following NCRT. This study delved into the modifications in the tumor microenvironment in patients who achieved PR before and after treatment. Our findings revealed heterogeneity in the tumor microenvironment between the pre- and post-treatment phases, suggesting that NCRT could potentially induce changes in the tumor microenvironment among LARC patients. Previous studies have shown that CRT can enhance the anti-tumor immune effect on the body [15, 28]. In our study, we observed a consistent increase in both CD4 + and CD8 + T cells following treatment in all the enrolled patients. This observation may be attributed to the release of tumor antigens through necrotic tumor cell death induced by CRT. Therefore, CRT could potentially facilitate the recruitment of CD8 + T cells by stimulating immune cells. CD4 + and CD8 + T cells play a major role in the anti-tumor immune response in humans. Kuwahara et al. [29] demonstrated that a high CD4 + T cell density in CRC was associated with improved relapse-free and disease-specific survival. Several reports have shown that high CD8 + T cell densities were associated with improved disease-free and overall survival, and that high-density CD8 + T cells were more likely to achieve complete remission [30, 31]. The increase in CD4 + and CD8 + T cells following treatment suggests that CRT may foster anti-tumor immunity by recruiting these T cell subtypes into tumor tissues, potentially leading to improved patient prognoses. The augmentation of anti-tumor immunity plays a pivotal role in enhancing the effectiveness of immune checkpoint inhibitors (ICIs). Notably, PD-1/PD-L1 ICIs primarily function by alleviating the suppression of CD8 + T cells, thereby boosting anti-tumor activity through their targeting of the PD-1/PD-L1 axis [32]. At present, classification of the tumor microenvironment using the PD-L1 status and tumor-infiltrating lymphocyte density has been put forth as a predictive marker for the response to ICIs. Patients with elevated levels of tumor-infiltrating lymphocytes and a positive PD-L1 status are more likely to benefit from ICIs [33]. After evaluating the expression of PD-1 and PD-L1, we observed that PD-1 and PD-L1 expression increased after NCRT, as in previous studies [34]. There is a suggestion that combining NCRT with ICIs may enhance the therapeutic effectiveness for patients who have achieved PR. Several clinical trials investigating the combination of CRT and immunotherapy for the treatment of rectal cancer are presently in progress, and some of these trials have shown promising and positive results [35]. We look forward to further exciting results and hope that this combined strategy will bring new advances to LARC treatment.

In our study, we observed that Tregs exhibited relative stability post-NCRT. Tregs, a specialized subset of T cells endowed with potent immunosuppressive capabilities, are often linked with a dismal prognosis in cases of malignant tumors [36]. Shinto et al. [28] conducted immunostaining for CD8 and Foxp3 on biopsy specimens obtained from 93 rectal cancer patients both before and after NCRT. Their findings revealed a significant increase in CD8 + T cells following treatment. Notably, the population of Foxp3 + Tregs showed no significant alteration, while a higher ratio of CD8 + T cells to Foxp3 + Tregs in the pre-treatment biopsies was associated with tumor regression. The same results were observed in our study. These outcomes suggest a favorable transformation within the tumor microenvironment subsequent to NCRT.

Apart from its impact on the adaptive immune system, NCRT also exerts influence on certain innate immune pathways, subsequently modulating T cell immune responses Neutrophils, recognized as the first responders to acute infections or inflammation [37], play a pivotal role in the tumor microenvironment. Within the tumor microenvironment, TANs have been implicated in promoting metastasis and influencing the clinical outcomes of CRC patients [38]. Our study revealed a slight increase in TANs following NCRT, implying a potential enhancement in their infiltration, possibly attributable to factors such as infection, and inflammation. Increased NK cell infiltration in CRC is generally thought to be associated with a better prognosis [39]. Preclinical studies have shown that low-dose radiation can boost NK cell infiltration within tumors, as demonstrated in a lung adenoma model [40]. In alignment with these findings, our investigation reported a post-NCRT increase in the number of CD56 + NK cells when compared to pre-NCRT levels. These results collectively suggest that NCRT may foster heightened NK cell infiltration within tumors, consequently augmenting the immune system’s ability to target and eliminate tumor cells.

In addition to the immunostimulatory effects, we also observed that CRT has specific immunosuppressive effects, which have the potential to counterbalance its immunostimulatory benefits. TAMs are essential components of the tumor microenvironment, and are strongly linked to the progression, metastasis, and chemotherapy resistance of colorectal tumors [6, 41]. Intriguingly, we observed an increase in TAMs post-treatment. This phenomenon may be attributed to the DNA damage, cell death, and heightened tumor hypoxia induced by the radiotherapy, leading to the upregulation of factors such as VEGF, SDF-1, and CSF-1, which in turn attract macrophages into the tumor environment [42]. Our findings align with previous research [43], confirming that CRT can indeed shape an immunosuppressive tumor microenvironment by bolstering TAM recruitment. Given these observations, we postulate that a combination of NCRT with therapies targeting TAMs could potentially enhance treatment efficacy. This idea is currently supported by some preclinical studies [11, 44]. Consequently, TAMs have emerged as a promising target for the treatment of LARC, warranting further in-depth investigation.

In this study, we observed a significant reduction in certain cell subsets after treatment, and the magnitude of this decrease exceeded the increase in cell subsets. EMT is a biological process through which epithelial cells undergo a phenotypic transformation into mesenchymal cells, and it is closely associated with invasion and metastasis [45]. To investigate the relationship between CRT and EMT in CRC patients, Kawamoto et al. [46] measured the expression levels of epithelial markers (CDH1), interstitial markers (VIM, FN1), and EMT-related transcription factors (SNAI1, SNAI2, TWIST1) in 26 rectal cancer patients pre- and post-NCRT. Their findings revealed a significant decrease in CDH1 expression after treatment (*p* = 0.0065), while VIM and FN1 expression significantly increased post-treatment (*p* < 0.0001 and *p* = 0.0002, respectively). Additionally, the EMT-related transcription factors also exhibited a significant increase after CRT. These results collectively support the notion that CRT can induce EMT in rectal cancer. On the contrary, we observed an increase in E-cadherin and CK levels after treatment, while vimentin, α-SMA, collagen I, and β-catenin exhibited significant decreases. This led us to tentatively propose that NCRT could potentially reverse the occurrence of EMT in patients who achieved PR, thus hampering the metastasis of rectal cancer. However, it is important to consider disparities in the irradiation regimen used. In the study of Kawamoto et al., patients received a short course of CRT (20 Gy/4f) along with 5-fluorouracil, uracil, and tegafur. In contrast, the patients in our study underwent long-term CRT (49.95–50.4 Gy/25–28f) with capecitabine as the concurrent chemotherapy. The differences in irradiation dosage may account for the variations in outcomes between the two studies.

Additionally, there is a paucity of studies regarding CD19 + B cells and CD14 + monocytes. Notably, in gastric cancer, CD19 + B cells have been shown to possess immunosuppressive properties and may potentially promote tumor progression [26], and peripheral blood monocytes are the primary source of TAMs. Our study indicated that NCRT could reduce CD19 + B cell infiltration and CD14 + monocytes in the tumor microenvironment.

In our current study, we utilized the Hyperion imaging system to provide initial insights into the specific effects of NCRT on the tumor microenvironment in patients who achieved PR. However, our study does have certain limitations. First, we observed a reduction in CD4 + CD45 + T cells. Nonetheless, since we did not differentiate between CD45RA + and CD45RO + subsets in this study, we lack precise information on how NCRT affects the initial CD4 + T cells and memory CD4 + T cell subsets. Further investigations are required to address this issue. Second, our study’s patient sample size is relatively small, and the Hyperion imaging system is expensive and challenging to apply to a broader spectrum of LARC patients. Consequently, increasing the sample size and conducting more comprehensive assessments of the tumor microenvironment changes before and after NCRT in PR patients with a cost-effective approach is crucial. This would help us gain a deeper understanding of the mechanisms underlying the relationship between NCRT and the tumor microenvironment in LARC patients who achieve PR after treatment, with the ultimate aim being to enhance the pCR rate.

## Conclusions

Our research reveals that in LARC patients who achieve a PR after NCRT, NCRT has a multifaceted impact on the tumor microenvironment. It is evident that NCRT’s influence goes beyond mere immune suppression or stimulation. Furthermore, CRT demonstrates the ability to reverse EMT, thereby hindering tumor metastasis. We hypothesize that NCRT primarily modifies the tumor microenvironment by significantly reducing the cellular components, potentially leading to adverse prognostic outcomes. In contrast, the expansion of immune cells stimulated by this therapeutic approach appears to be modest. These findings provide a valuable theoretical foundation for the utilization of NCRT in combination with ICIs or targeted therapies in the management of rectal cancer. This synergistic approach has the potential to enhance the likelihood of achieving a pCR in a greater number of PR patients, thereby improving their clinical outcomes.

## Supplementary Information

Below is the link to the electronic supplementary material.

Supplementary file1 (DOCX 25 KB)

## Data Availability

All data generated or analyzed during this study are included in this article. Further inquiries can be directed to the corresponding author.
