# Supplementary material for: Analysis of tumor microenvironment alterations in partially responsive rectal cancer patients treated with neoadjuvant chemoradiotherapy
Source: Int J Colorectal Dis. 2024 Jun 26;39(1):99. doi: 10.1007/s00384-024-04672-1 (PMC11208236; doi:10.1007/s00384-024-04672-1)
Supplement: Supplementary file 1 — Supplementary file1 (DOCX 25 KB) [file 384_2024_4672_MOESM1_ESM.docx]

**Supplementary Table 1**. Antibodies targeting the tumor immune environment (TME) of locally advanced rectal cancer (LARC)

| Label | Antibody | Vendor | Catalog | Clone |
| --- | --- | --- | --- | --- |
| Dy163 | CD45 | ebioscience | 14-9457-82 | 2B11 |
| Er170 | CD3 | Fluidigm | 3170019D | Polyclonal, C-Termina |
| Gd156 | CD4 | Fluidigm | 3156033D | EPR6855 |
| Gd155 | Foxp3 | Biolegend | 320102 | 206D |
| Dy162 | CD8a | Fluidigm | 3162035D | D8A8Y |
| Sm149 | CD11b/Mac-1 | Fluidigm | 3149028D | EPR1344 |
| Sm154 | CD11c | Fluidigm | 3154025D | Polyclonal |
| Nd144 | CD14 | Fluidigm | 3144025D | EPR3653 |
| Eu153 | CD15 | Biolegend | 301902 | HI98 |
| Nd146 | CD16 | Fluidigm | 3146020D | EPR16784 |
| Nd142 | CD19 | Fluidigm | 3142014D | 6OMP31 |
| Nd145 | CD33 | Fluidigm | 3145017D | Polyclonal |
| Sm152 | CD56 | Abcam | 9018 | RNL-1 |
| Tb159 | CD68 | Biolegend | 916104 | KP1 |
| Sm147 | IFN-γ | Abcam | 9657 | Polyclonal |
| Yb174 | HLA-DR | Fluidigm | 3174023D | YE2/36 HLK |
| Nd150 | CD274/PD-L1 | Fluidigm | 3150031D | E1L3N |
| Yb172 | CD273/PD-L2 | Fluidigm | 3172028D | 176611 |
| Ho165 | CD279/PD-1 | Fluidigm | 3165039D | EPR4877(2) |
| Dy164 | CK AE1/AE3 | Biolegend | 914204 | AE-1/AE-3 |
| Pr141 | α-SMA | ebioscience | 14-9760-82 | 1A4 |
| Tm169 | collagen I | Abcam | 88147 | 3G3 |
| Gd158 | CD324/E-Cadherin | Abcam | 231303 | 4A2 |
| Nd148 | Vimentin | Abcam | 8978 | RV202 |
| Er168 | Ki-67 | Biolegend | 350502 | Ki-67 |
| Lu175 | b-catenin | Biolegend | 844603 | 12F7 |

**Supplementary Table 2**. Significance test of 26 kinds of antibodies pre- and post-treatment

| [Antibody](javascript:;) | | Mean（before） | Mean （after） | P-value | Change of antibody | Significance |
| --- | --- | --- | --- | --- | --- | --- |
| CD4 | 0.0433 | 0.0812 | 6E-41 | up | significant |  |
| CD15 | 0.0959 | 0.7705 | 2E-256 | up | significant |  |
| PD_1 | 0.014 | 0.016 | 3E-07 | up | significant |  |
| PD_L1 | 0.0385 | 0.0437 | 6E-09 | up | significant |  |
| CK_AE1AE3 | 0.0818 | 0.2568 | 4E-191 | up | significant |  |
| CD16 | 0.0225 | 0.0504 | 2E-212 | up | significant |  |
| E_Cadherin | 0.0502 | 0.3093 | 6E-284 | up | significant |  |
| CD11b | 0.2228 | 0.4752 | 4E-133 | up | significant |  |
| CD11c | 0.048 | 0.1235 | 1E-203 | up | significant |  |
| CD19 | 0.0461 | 0.0448 | 2E-01 | down | not significant |  |
| b_Catenin | 0.718 | 0.8762 | 1E-12 | up | significant |  |
| Foxp3 | 0.0185 | 0.032 | 3E-12 | up | significant |  |
| CD68 | 0.3406 | 0.7411 | 2E-129 | up | significant |  |
| aSMA | 0.0795 | 0.1211 | 2E-18 | up | significant |  |
| PD_L2 | 0.0573 | 0.055 | 1E-02 | down | not significant |  |
| HLA_DR | 0.3575 | 0.7294 | 1E-101 | up | significant |  |
| CD56 | 0.0369 | 0.0627 | 4E-71 | up | significant |  |
| CD33 | 0.0367 | 0.0415 | 2E-09 | up | significant |  |
| CD14 | 0.1908 | 0.2975 | 9E-107 | up | significant |  |
| CD8 | 0.0326 | 0.3442 | 1E-278 | up | significant |  |
| CD3 | 0.0515 | 0.1414 | 1E-210 | up | significant |  |
| CD45 | 0.186 | 0.4246 | 7E-268 | up | significant |  |
| vimentin | 0.3254 | 0.4042 | 3E-19 | up | significant |  |
| IFN_g | 0.0837 | 0.1872 | 2E-42 | up | significant |  |
| collagen1 | 0.1048 | 0.2649 | 2E-137 | up | significant |  |
| Ki67 | 0.1107 | 0.2193 | 3E-41 | up | significant |  |

**Supplementary Table 3** Proportion of changes in all cell subsets pre- and post-treatment

| Cell subsets | Proportion pre-NCRT | | Proportion post-NCRT | Fold change |
| --- | --- | --- | --- | --- |
| S1 | | 0.005945704 | 0.000174672 | 34.03915356 |
| S10 | | 0.119477048 | 0.037030863 | 3.22641819 |
| S11 | | 0.033757138 | 0.003310454 | 10.19713112 |
| S12 | | 0.069635005 | 0.007622637 | 9.135290178 |
| S13 | | 0.003028259 | 0.009456945 | 0.320215377 |
| S14 | | 0.008394836 | 0.04099274 | 0.204788355 |
| S15 | | 0.028834038 | 0.036948518 | 0.780384157 |
| S16 | | 0.007279892 | 0.042553683 | 0.171075478 |
| S17 | | 0.018462677 | 0.040076269 | 0.460688515 |
| S18 | | 0.058624735 | 0.033893168 | 1.729691794 |
| S19 | | 0 | 0.00349345 | 0 |
| S2 | | 0.013740838 | 0.017257186 | 0.796238647 |
| S20 | | 0.006244541 | 0.016950457 | 0.368399575 |
| S21 | | 0.063330198 | 0.044775441 | 1.414395846 |
| S22 | | 7.94E-05 | 0.044041792 | 0.00180383 |
| S23 | | 0 | 0.009451402 | 0 |
| S24 | | 0.005367982 | 0.050575104 | 0.106138821 |
| S25 | | 0.05738759 | 0.045853096 | 1.251553218 |
| S26 | | 0.037431381 | 0.014594505 | 2.564758469 |
| S27 | | 0.053407467 | 0.035240223 | 1.515525775 |
| S28 | | 0.009978095 | 0.003753487 | 2.658353283 |
| S29 | | 0.019919249 | 0.010396403 | 1.915975125 |
| S3 | | 0.023394616 | 0.043861447 | 0.53337538 |
| S30 | | 0.034405281 | 0.032283931 | 1.065709177 |
| S31 | | 0.028446823 | 0.011804857 | 2.409755909 |
| S32 | | 0.018186176 | 0.028268398 | 0.643339464 |
| S33 | | 0.053960163 | 0.035388378 | 1.524798981 |
| S34 | | 0.021657 | 0.080595624 | 0.268711865 |
| S35 | | 0.086306278 | 0.054205567 | 1.592203221 |
| S36 | | 0.016265771 | 0.014469914 | 1.124109734 |
| S37 | | 0.040554581 | 0.035747389 | 1.134476726 |
| S4 | | 0.003609496 | 0.003286792 | 1.098181974 |
| S5 | | 0.001992146 | 0.006343761 | 0.314032344 |
| S6 | | 0 | 0.013441542 | 0 |
| S7 | | 0.014298214 | 0.024292208 | 0.588592622 |
| S8 | | 0.000135324 | 0.034898022 | 0.003877705 |
| S9 | | 0.036462014 | 0.032669676 | 1.116081274 |
